# Evaluation of a large language model (ChatGPT) versus human researchers in assessing risk-of-bias and community engagement levels: a systematic review use-case analysis

**DOI:** 10.1093/eurpub/ckaf072

**Published:** 2025-06-10

**Authors:** Marcello Di Pumpo, Maria Teresa Riccardi, Vittorio De Vita, Gianfranco Damiani

**Affiliations:** Department of Life Sciences and Public Health, Università Cattolica del Sacro Cuore, Rome, Italy; Italian Society for Artificial Intelligence in Medicine (SIIAM—Società Italiana Intelligenza Artificiale in Medicina), Rome, Italy; Cancer Screening Unit, Local Health Unit Roma 2, Rome, Italy; Department of Life Sciences and Public Health, Università Cattolica del Sacro Cuore, Rome, Italy; Department of Life Sciences and Public Health, Università Cattolica del Sacro Cuore, Rome, Italy; Department of Woman and Child Health and Public Health, Fondazione Policlinico Universitario A. Gemelli IRCCS, Rome, Italy

## Abstract

Large language models (LLMs) like OpenAI’s ChatGPT (generative pretrained transformers) offer great benefits to systematic review production and quality assessment. A careful assessment and comparison with standard practice is highly needed. Two custom GPTs models were developed to compare a LLM’s performance in “Risk-of-bias (ROB)” assessment and “Levels of engagement reached (LOER)” classification vs human judgments. Inter-rater agreement was calculated. ROB GPT classified a slightly higher “low risk” overall judgments (27.8% vs 22.2%) and “some concern” (58.3% vs 52.8%) than the research team, for whom “high risk” judgments were double (25.0% vs 13.9%). The research team classified slightly higher “low risk” total judgments (59.7% vs 55.1%) and almost double “high risk” (11.1% vs 5.6%) compared to “ROB GPT” (55.1%), which rated higher “some concerns” (39.4% vs 29.2%) (*P* = .366). With regards to LOER analysis, 91.7% vs 25.0% were classified “Collaborate” level, 5.6% vs 61.1% as “Shared leadership”, and 2.8% as “Involve” vs 13.9% by researchers, while no studies classified in the first two engagement level vs 8.3% and 13.9%, respectively, by researchers (*P* = .169). A mixed-effect ordinal logistic regression showed an odds ratio (OR) = 0.97 [95% confidence interval (CI) 0.647–1.446, *P* = .874] for ROB and an OR = 1.00 (95% CI = 0.397–2.543, *P* = .992) for LOER compared to researchers. Partial agreement on some judgments was observed. Further evaluation of these promising tools is needed to enable their effective yet reliable introduction in scientific practice.

## Introduction

Systematic reviews synthesizing evidence of the effectiveness of interventions are a cornerstone of clinical guidelines and evidence-based medicine [1]. These studies play a crucial role in shaping public health evidence-based practice, providing the foundation for informed decision-making [1]. Quality assessment is a key part in building a high-quality systematic review, and evaluating risk of bias (ROB) is an essential element of such studies [2]. Methods for evaluating ROB have evolved from study quality checklists to an increasing focus on factors that affect the internal validity of the results of studies [3]. However, as recently reported [3], these processes can be inherently resource-intensive, demanding significant time, expertise, and financial investments [3]. The advent of artificial intelligence (AI) in scientific practice, and in particular of large language models (LLMs), has introduced great potential for better resource management and innovation. In November 2022, OpenAI publicly launched ChatGPT (generative pretrained transformers), a novel open-source LLM, reaching over a hundred million users in the first months. These AI models have already proven useful in a myriad of health care-related tasks and processes [4]. Carobene *et al.* [5] shed light on the increasing adoption of AI in scientific publishing, emphasizing the imperative of evaluating its various roles, associated risks, and ethical implications in depth. In particular, many authors have already provided evidence on the specific benefits of integrating these new tools in the systematic review production process [6]. AI can potentially support all phases of systematic reviews [7–11]. However, careful consideration as to how, in which phase and which specific AI-based tools to employ is necessary [12]. Despite its benefits, its implementation requires blending its potential with researchers’ expertise and avoiding uninformed overreliance. A balanced strategy entailing AI integration with independent critical thinking is in fact needed [13]. The reliability of these tools has profound scientific and ethical implications.

There are to date some studies that tested commonly available LLMs in assessing quality and synthesizing scientific evidence against current standard scientific practice performed solely by human researchers. Lai *et al.* [14] tested the capability of ChatGPT and Claude to assess the ROB in randomized clinical trials (RCTs). They compared the performance of these LLMs against the current standard practice of ROB assessment performed by human experts. The study reported how LLMs demonstrate substantial potential as supportive tools in the systematic review processes, achieving high accuracy and consistency in their assessments compared to human reviewers. Two other studies [15, 16] evaluated the performance of RobotReviewer, a machine learning system designed for semiautomated risk-of-bias (ROB) assessment in RCTs within nursing-related Cochrane reviews, with the aim to compare its reliability and accuracy against human reviewers’ judgment. One study [15] showed how RobotReviewer’s reliability with human reviewers was similar for most domains and better for allocation concealment, blinding of participants and personnel, and overall risk of bias. The other article [16] reported how RobotReviewer yielded a moderate degree of agreement with human reviewers for randomization and allocation concealment, and an adequate sensitivity for detecting low risk of selection bias, when tested on RCTs included in nursing-related Cochrane reviews.

There is however not a fair amount of evidence to support the validity and accuracy of these promising tools and not many different research fields on which they have been tested so far.

The current study aims therefore to validate the judgments of an *ad hoc* customized OpenAI’s GPT, developed with a no-code approach programming skills prerequisites and therefore accessible to nontechnical researchers, through a comparison of its performance in ROB assessment and levels of engagement reached (LOER—by communities under study) evaluation against the classification performed in a reference published article from the public health field.

The rationale for this design choice is in line with similar studies and stems from the increasing interest in leveraging AI-driven tools to automate and enhance evidence synthesis while ensuring methodological rigor and reproducibility. A systematic review was chosen as a use case as this study design requires consistent and precise classification of studies based on predefined inclusion/exclusion criteria, serving therefore as a structured and suitable example for evaluating the capabilities of LLMs in handling complex conceptual judgment tasks.

The goal is to assess the potential of an accessible customized LLM tool to improve the efficiency of a traditionally labor-intensive systematic review task while maintaining the rigor of human-led classification. By demonstrating its ability to streamline the review process, reduce researcher workload, and enhance accessibility, this study could help advance methodological innovation, providing the research community with a scalable and reliable tool.

## Methods

### Study design

The present study is a methodological study by design as defined by Khalil [17], focusing on the validation of AI-driven assessments in the context of ROB and LOER assessment process of a systematic review.

### Preliminary data preparation

Before applying LLMs to the systematic review classification task, we conducted a structured data collection and preparation process to ensure the validity and reliability of our evaluation. First, we sourced researchers’ responses by selecting a systematic review dataset that required conceptual classification of studies based on predefined inclusion/exclusion criteria. These classifications were originally conducted by domain experts following rigorous screening protocols, ensuring a high-quality benchmark for comparison. To enhance reproducibility, we documented the criteria used by human reviewers and ensured that their decision rationales were explicitly recorded.

Once the expert-classified dataset was compiled, we prepared the data for LLM processing. This involved standardizing text inputs by structuring study descriptions, ensuring consistency in formatting, and anonymizing any sensitive information if necessary. Additionally, we developed a prompting strategy to guide the LLMs, incorporating explicit instructions that mirrored the human decision-making framework. To account for potential variability in LLM responses, we implemented double-repetition verification, where each classification was performed twice to assess consistency. Finally, we preprocessed the data to enable structured statistical analysis, categorizing classification outputs into an ordinal scale for subsequent evaluation using a mixed-effect ordinal logistic regression, with random effects for the different selected studies from the systematic review.

By meticulously preparing the dataset and aligning LLM prompts with human decision-making logic, we ensured that our analysis reflected a realistic, practice-oriented assessment of LLMs’ capability in assisting systematic reviews.

### Custom GPT setup

Two custom GPTs were generated and named respectively “ROB GPT” for Rob assessment and “LOER GPT” for the assessment of the LOER by each study, using a no-code approach. This approach consists in a development paradigm that allows users to modify and tailor generative models, such as GPT, using intuitive, graphical interfaces without the need for traditional programming. This enables nontechnical users to adjust model parameters, behaviors, and outputs to meet specific domain requirements or interaction contexts, facilitating widespread accessibility and rapid deployment of artificial intelligence solutions. It was chosen for its accessibility and efficiency, enabling the rapid configuration and deployment of the AI model without the need for extensive programming expertise [18].

The details regarding customization, prompting development and ChatGPT interaction are available in Supplementary File S1. The Custom GPT was created using the ChatGPT-4 subscription-based version available in February 2024, which was the only version used.

The paper by Riccardi *et al.* [19] as a reference paper for the study was based on several crucial factors. A first reason is the expertise of the researchers on the topic and methodology, considering they themselves performed the original classification, to minimize bias during the re-evaluation phase and enable appropriate discussion. Secondly, the chosen study was deemed highly relevant for evaluating the Custom GPT’s capacity for critical thinking, given the complex topic of engagement of underserved communities and how public health interventions differ substantially from biomedical studies and clinical interventions [1]. Another significant reason for selecting this study was that only RCTs were included, ensuring a uniform methodological framework across all analyzed papers. Uniformity in methodology was also ensured by the sole use of the Cochrane ROB-2 tool, thereby streamlining the evaluation process and enhancing comparability. A final reason consists in the fact that the authors performed a complex task of LOER classification, following CBPR methodology by McCloskey *et al.* (1: Outreach: researchers provide information to community; 2: Consult: communication flows to the community and then back; 3: Involve: there is a participatory form of communication and entities cooperate with each other; 4: Collaborate: there is a partnership with community on each aspect of project; 5: Shared leadership: the final decision-making is at community level) [20]. The details of each primary study in the reference study and relative ROB and LOER judgment are available as Supplementary File S2.

### Statistical analysis

Descriptive statistics were used to summarize the ordinal categorical variable “judgement” as frequencies and percentages, providing an overview of the distribution of key classification categories. The comparison between ChatGPT and human raters was performed by calculating inter-rater agreement (IRA) between two raters’ overall judgments using the Cohen’ kappa statistic with linear weighting (e.g. LR vs SC, with 0.50 as assigned weight compared to 1.0 for LR vs HR), adapting the methodology from Hartling *et al.* [21], which accounts for the ordinal nature of the categories: “Low Risk,” “Some Concern,” and “High Risk.” Cohen’s kappa is an IRA measure that quantifies the consistency between two raters while accounting for agreement occurring by chance. Unlike simple percent agreement, kappa adjusts for random chance, making it a more robust measure for assessing classification reliability, especially with ordinal outcomes. An ordinal logistic regression was performed to obtain effect size measures, reported as odds ratio (OR) and 95% confidence interval (CI). Statistical analysis was conducted using STATA software [StataCorp (2019) Stata Statistical Software: Release 16. StataCorp LLC, College Station, TX, USA].

### Ethical issues

This systematic review did not require ethical approval as it involved the secondary analysis of existing published data, with no direct interaction with human participants or the use of personal or confidential data. All data sources included in this review are publicly available and were used in full compliance with ethical standards.

## Results

All 36 primary studies present systematic review from the reference study were included in the analysis. A total of 216 judgments, considering five judgments and the overall ROB judgment for each study, were generated and compared as previously described.

### Data regarding ChatGPT and human judgment are presented in Supplementary File S3

#### ROB analysis

With regards to the 36 overall ROB judgments, 17 agreements (47.2%) were observed between ROB GPT and the research team.

When considering all ROB judgments, a total of 128 (59.3%) agreements out of 216 judgments were recorded. In detail, for 40.3% of the judgments (*n* = 87), a disagreement of one degree between ChatGPT and the research team was found, while for 3.7% (*n* = 8) a disagreement of two degrees (LR vs HR, 1.00 assigned weight) was observed for six studies in total (DeHaven 2011 Froelicher 2011, Masi 2003, Schoenberg 2017, Tanjasiri 2015, Zoellner 2011) as shown in Supplementary File S3.

Regarding overall judgments, as shown in Table 1, ROB GPT classified a slightly higher percentage of studies as ‘“low risk” (27.8% vs 22.2%) and “some concern” (58.3% vs 52.8%), while “high risk” judgments were more than double for the research team compared with ROB GPT (25.0% vs 13.9%).

**Table 1.** Comparison of GPT and human categories by ROB level for overall and total judgments.

Category	GPT overall—*N* (%)	RES overall—*N* (%)	GPT total—*N* (%)	RES total—*N* (%)
Low risk	10 (27.8)	8 (22.2)	119 (55.1)	129 (59.7)
Some concern	21 (58.3)	19 (52.8)	85 (39.4)	63 (29.2)
High risk	5 (13.9)	9 (25.0)	12 (5.6)	24 (11.1)
Total	36 (100)	36 (100)	216 (100)	216 (100)

RES, research team.

Similar results are found for total judgments, where the research team classified a slightly higher percentage of studies as “low risk” (59.7% vs 55.1%) and almost double percentage of studies as “high risk” (11.1% vs 5.6%) compared to “ROB GPT” (55.1%), which rated a significantly higher percentage of studies as having “some concerns” (39.4%) compared to the research team (29.2%).

As shown in Table 2, with regards to IRA inferential analysis for ROB judgments, for the overall domain, Cohen’s kappa 0.0455 indicates low agreement level (*P* = .366).

**Table 2.** Cohen’s kappa IRA of ROB and LOER judgments analysis.

Judgment	Kappa value	*P*-value
ROB Domain 1	0.1641	.1248
ROB Domain 2	−0.1928	.9154
ROB Domain 3	0.0676	.2883
ROB Domain 4	0.1616	.1178
ROB Domain 5	0.2105	.0915
Overall ROB	0.0455	.3655
LOER	0.0445	.1497

The mixed-effect ordinal logistic regression performed showed an OR = 0.97 (95% CI 0.647–1.446, *P* = .874).

#### LOER analysis

Regarding LOER analysis, 10 (27.8%) agreements were observed between the two raters. With regards to LOER analysis, as shown in Table 3, 91.7% vs 25.0% studies are classified at the “Collaborate” level, 5.6% vs 61.1% as “Shared leadership” and only one study (2.8%) as “Involve” vs 13.9% as classified by the research team, while no studies classified in the first two engagement level vs 8.3% and 13.9% as classified by the research team.

**Table 3.** LOER analysis comparison between ChatGPT and the Research Team (RES)

Category	LOER GPT (%)	LOER RES (%)
Outreach	–	3 (8.3)
Consult	–	5 (13.9)
Involve	1 (2.8)	5 (13.9)
Collaborate	33 (91.7)	9 (25.0)
Shared leadership	2 (5.6)	14 (61.1)
Total	36 (100)	36 (100)

The IRA analysis for LOER judgments also shows a modest agreement with no statistical significance is also documented (*P* = .169).

The mixed-effect ordinal logistic regression performed showed has OR = 1.00 (95% CI = 0.397–2.543, *P* = .992).

## Discussion

The results show how for overall and single-domain ROB and LOER judgments some level of agreement, higher for total than overall judgments, with however no statistical significance found.

A first consideration regards how high-risk classification rate by the research team was higher (double for total judgments) compared to ROB GPT. This could be indicative of how human evaluators might have a deeper understanding of nuanced details in textual analysis, allowing for a more thorough and critical classification of study quality [22]. Another possibility is that the “alignment” process toward ethical values and nondiscriminatory contents of the output in generative AI applications in later versions [23] has progressively rendered these models generally less prone to overcritical and disqualifying judgments [22].

Another important consideration regards the difference in “some concern” classification, with ROB GPT classifying almost 10% more total judgments than the research team (less for overall). This could potentially be due to how, avoiding high-risk judgment, the customized GPT might relegate a larger number of studies to the immediate lower risk category due to detected ambiguities or minor issues that prevent it from being classified as low risk. However, another important explanation could be that a less discriminative tool could suffer from what is known as “central tendency bias” [24] when confronted with complex classification tasks in a multiple-answer scenario [25]. However, no difference resulted statistically significant.

A third consideration is how the research team identified a similar percentage of studies as low risk compared to ROB GPT, (59.72% vs 55.09% for total, 22.2% vs 27.8% for overall) indicating a higher concordance when low-risk studies are examined, possibly because properly reported high-quality features are easier to classify for both raters, compared to identifying what is missing. For studies that are high-quality as presenting all necessary items with appropriate design and reporting, LLMs appear therefore more able to match human classification abilities. No difference, however, resulted statistically significant.

A fourth consideration regards the LOER classification. LOERGPT’s judgments are skewed toward higher engagement levels and in most cases corresponding to the “Collaborate” engagement level. It is evident how the research team presents a wider variety of judgment choices, with almost 50% discordant judgments with respect to LOER GPT. This is possibly due to the previously mentioned higher discriminative capacity of human raters [22] more evident with this complex conceptual classification task rather than the ROB classification, being the latter more standardized and algorithmic in nature. Also, considering how higher engagement judgments are produced by LOER GPT, the “alignment” phenomenon previously described [23] could still be considered among the causes. In addition, the observed difference could be partially explainable with central tendency bias [24], even though acknowledging how “Collaborate” is not the exact central category in the judgments’ distribution.

The study findings, therefore, resonate with similar recent studies present in literature [14–16]. These underscore how researchers could in future refer to AI automation, keeping responsible supervision, for some tasks with well-reported material and objectives, while combining machine efficiency with human judgment to achieve a more robust and nuanced quality assessment process in case of complex and multilevel judgments. In this regard, further calibration of AI models and training of researchers will be needed to improve consistency and effective integration in everyday public health scientific practice.

On a final note, neither agreement nor disagreement resulted statistically significant. LLMs are indeed different in logical reasoning and output from human evaluators, however provided are generally in line judgments with humans in many cases. It is important to acknowledge the potential of these promising tools and generate significant evidence on them from a scientific standpoint [26–28].

### Study limitations and strengths

This study has some limitations and strengths.

A key limitation is that the sample size, however not minimal, could have contributed to not reaching statistical significance. However, the choice of the reference study was detailed in the Methods section and, considering the exploratory nature of the study, the research team believes it is a good first choice to pave the way for further analysis with greater primary data numbers.

A major strength relies in particular in the LOER analysis, because it entailed complex conceptual classification tasks, which cannot be reduced to algorithmic logic but needs a synthetic and multidimensional assessment of the study setting, intervention, and stakeholders involved.

Another limitation consists in the fact that conversational LLMs might fall short in reproducibility when producing output. This is a widely documented phenomenon, known as “output variance” or “non-deterministic behaviour” and it is due to: sampling strategies, LLMs often use probabilistic sampling methods which introduces randomness; model architecture, transformer-based models have inherent stochasticity in their attention mechanisms; training data and training methodology. The observed variance allows for creativity and diverse responses but can also lead to inconsistency in applications requiring deterministic outputs [29, 30]. To assess stability and consistency for our study, prompting was repeated twice per each classification, and outputs were compared. While slight phrasing variations were noted, core conclusions and judgment always remained equal, suggesting robustness and stability in the customized GPTs reasoning across prompts. The decision not to enforce strict standardization, by manually implementing control of hyperparameters like temperature and seed by use of application programming interface, was in line with the goal of evaluating easy-to-implement solutions for all researchers not expert in generative AI use and technicalities. Future validation works will need to balance the need for standardization and reproducibility with technology accessibility and ease of deployment for nontechnical users.

Lastly, a no-code approach was chosen instead of delving into the building and validation of complex code-based architectures. This approach requires in fact no major technical training in the specific field and can be easily used and reproduced by a wide array of researchers from different fields. However, positive in terms of accessibility and scalability, its comparative effectiveness to other approaches requiring technical skills needs further evaluation by future studies.

## Conclusions

LLMs are very promising tools, able to imitate human reasoning at an unprecedented level, and are being increasingly and rapidly adopted by researchers worldwide. This is among the first works to assess an AI-based automated approach to systematic reviews quality assessment feasible in the public health field to support healthcare professionals and researchers in ROB assessment and related conceptual automated analysis for systematic reviews execution achieving an efficient while reliable automated ROB classification method is in fact key for sustainable high-quality research synthesis development.

The specific added value for the public health field stems from the application of an AI-based tool to a complex task like LOER classification, which requires a multidimensional evaluation of a public health approach at the community level.

There is an urgent need inside the scientific community to evaluate AI-based tools to provide accurate suggestions for their appropriate use and progressive integration in everyday scientific practice. Further studies experimenting on multiple different tasks with appropriate sample sizes and methodology are needed.

## Supplementary Material

ckaf072_Supplementary_Data

## Data Availability

All data sources included in this review are publicly available in the reference published article by Riccardi *et al*. Key points Large language models (LLMs) like OpenAI’s ChatGPT (generative pretrained transformers) offer great benefits to systematic review production and quality assessment. Despite its benefits, its implementation requires blending its potential with researchers’ expertise and avoiding uninformed overreliance. A careful assessment and comparison with standard practice is highly needed. Two custom GPTs models were developed to compare a LLM’s performance in “Risk-of-bias (ROB)” assessment and “Levels of engagement reached (LOER)” classification vs the research team’s from a selected article. The results show how for overall and single-domain ROB and LOER judgments some level of agreement is observed, higher for total rather than overall judgments. Further evaluation of these promising tools is needed to enable their responsible and efficient introduction in scientific practice, in combination with human researchers’ skills and experience.
