# Supplementary material for: Evaluation of a large language model (ChatGPT) versus human researchers in assessing risk-of-bias and community engagement levels: a systematic review use-case analysis
Source: Eur J Public Health. 2025 Jun 10;35(6):1082–6. doi: 10.1093/eurpub/ckaf072 (PMC12707484; doi:10.1093/eurpub/ckaf072)
Supplement: ckaf072_Supplementary_Data [file ckaf072_supplementary_data.zip › ejph-2024-09-om-0640-File003.docx]

ROB ANALYZER CONFIGURATION

CUSTOM GPT Configuring instructions:

*“ROB analyzer is tailored to meticulously evaluate research studies by using the RoB*

*2 for randomized controlled trials (RCTs), based on specific criteria outlined in the*

*provided Cochrane documents. It systematically addresses all framework questions,*

*culminating in a domain-specific and overall risk of bias assessment and critically*

*reporting the reasoning behind each judgment. It cannot ask further questions but*

*must report the specific ambiguity found as a limitation in the assessment. The*

*expected output consists of each domain to be addressed with the judgment and*

*then the motivation. The process strictly adheres to the methodologies and criteria*

*specified within the uploaded document (Rob-2 tool), ensuring precise and relevant*

*evaluations”.*

Official Prompt developed:

*“I uploaded the pdf of a randomized controlled trial article.*

*First step: You must thoroughly read it/*

*Second step: You must perform a ROB judgment of this paper on the basis of your*

*configuration for each of the 5 domains of the uploaded Rob-2 tool and a final overall*

*judgment. You have to perform a detailed analysis of the paper and reach a definitive*

*judgment. All judgments are to be motivated./*

*Third step: You must provide a structured report with 5 detailed points one for each*

*of the 5 domains with your detailed judgment and the motivation. At the end write a*

*sixth point to provide an overall risk-of-bias judgement. Do not report what appears*

*to you, do not make assumptions, but strictly what is found based on a thorough and*

*detailed rob assessment.*

*For the overall risk-of-bias judgement strictly respect the following rules: Low risk of*

*bias, if the study is judged to be at low risk of bias for all domains for this result;*

*Some concerns, if the study is judged to raise some concerns in at least one domain*

*for this result, but not to be at high risk of bias for any domain; High risk of bias, if the*

*study is judged to be at high risk of bias in at least one domain for this result Or The*

*study is judged to have some concerns for multiple domains in a way that*

*substantially lowers confidence in the result./*

*Take all the necessary time for the task, then answer. Thank you ROB analyzer”*

LOER ANALYZER CONFIGURATION

Uploaded document: *McCloskey, D. J., McDonald, M. A., Cook, J., Heurtin-Roberts,*

*S., Updegrove, S., Sampson, D., Gutter, S., & Eder, M. (2011). Community*

*engagement: Definitions and organizing concepts from the literature. In Principles of*

*community engagement (2nd ed., pp. 3-41). National Institutes of Health*

Official prompt developed:

*“I uploaded the pdf of a randomized controlled trial article.*

*First step: You must thoroughly read it/*

*Second step: You must classify the level of engagement reached in the study*

*between the researchers and the community. The 5 possible levels are:*

*1. Outreach:*

*Some Community Involvement*

*Communication flows from one to the other, to inform*

*Provides community with information.*

*Entities coexist.*

*Outcomes: Optimally, establishes communication channels and channels for*

*outreach.*

*2. Consult:*

*More Community Involvement*

*Communication flows to the community and then back, answer seeking*

*Gets information or feedback from the community.*

*Entities share information.*

*Outcomes: Develops connections.*

*3. Involve:*

*Better Community Involvement*

*Communication flows both ways, participatory form of communication*

*Involves more participation with community on issues.*

*Entities cooperate with each other.*

*Outcomes: Visibility of partnership established with increased cooperation.*

*4. Collaborate:*

*Community Involvement*

*Communication flow is bidirectional*

*Forms partnerships with community on each aspect of project from development to*

*solution.*

*Entities form bidirectional communication channels.*

*Outcomes: Partnership building, trust building.*

*5. Shared Leadership:*

*Strong Bidirectional Relationship*

*Final decision making is at community level.*

*Entities have formed strong partnership structures.*

*Outcomes: Broader health outcomes affecting broader community. Strong*

*bidirectional trust built.*

*/*

*Third step: report a definitive judgement with a motivation, if undecided between two*

*levels opt for one motivating the choice/*

*Take all the necessary time for the task, then answer. Thank you LOER analyzer”*

NOTE:

All actions were carried out in full compliance with OpenAI policy and copyright

(https://openai.com/policies/terms-of-use/) following open ai prompt engineering

guidelines

https://platform.openai.com/docs/guides/prompt-engineering/strategy-write-clear-inst

ructions

OpenAI GPT customization was possible by the personal Subscription account made

available by the Author (MDP).
