# Supplementary material for: Evaluation of a large language model (ChatGPT) versus human researchers in assessing risk-of-bias and community engagement levels: a systematic review use-case analysis
Source: Eur J Public Health. 2025 Jun 10;35(6):1082–6. doi: 10.1093/eurpub/ckaf072 (PMC12707484; doi:10.1093/eurpub/ckaf072)
Supplement: ckaf072_Supplementary_Data [file ckaf072_supplementary_data.zip › ejph-2024-09-om-0640-File004.docx]

| **Author, Year** | **Brief Description** | **Levels of Engagement** |
| --- | --- | --- |
| Andrews, 2016, USA | African American women living in subsidized government housing with high smoking prevalence. | Collaborate |
| Angell, 2003, USA | Women with primary breast cancer living in rural and isolated communities in California, addressing breast cancer survivorship. | Collaborate |
| Celano, 2012, USA | Low-income African American children and their families facing high asthma morbidity and mortality rates. | Outreach |
| Choi, 2016, USA | American Indians/Alaska Natives living in rural or reservation-based areas with the highest smoking prevalence among any racial/ethnic group in the U.S. | Shared Leadership |
| DeHaven, 2011, USA | African Americans living in areas with high poverty, female-headed households, and unemployed males, experiencing higher morbidity and mortality rates. | Shared Leadership |
| Derose, 2014, USA | Residents of Los Angeles evaluated for park-to-citizen ratio and its impact on health outcomes. | Shared Leadership |
| Froelicher, 2011, USA | Low-income African American smokers facing higher age-adjusted mortality rates from tobacco-related cancers and cardiovascular disease. | Consult |
| Goldfinger, 2012, USA | Black and Latino populations impacted by stroke with suboptimal stroke recurrence rates and prevention strategies. | Shared Leadership |
| Ingraham, 2017, USA | Overweight or obese lesbian and bisexual women aged 40 and older with higher body weight than heterosexual women, addressing obesity and mindfulness. | Shared Leadership |
| Islam, 2013, USA | NYC Korean population with a higher risk of diabetes compared to the general population, focusing on diabetes prevention. | Shared Leadership |
| Jernigan, 2018, USA | Native American communities from Chickasaw and Choctaw Nations with obesity, diabetes, and hypertension exceeding national averages. | Consult |
| Kaholokula, 2012, USA | Pacific Islanders with high obesity prevalence and associated health risks. | Shared Leadership |
| Kneipp, 2012, USA | Women in the Temporary Assistance for Needy Families (TANF) program experiencing higher rates of depressive disorders, PTSD, and poor general health. | Collaborate |
| Larkey, 2012, USA | Multicultural and underinsured individuals with low participation in cancer prevention research programs. | Collaborate |
| Lee, 2014, USA | Vietnamese Americans facing high incidence of colorectal cancer and low screening rates, addressing cancer prevention. | Shared Leadership |
| Ma, 2018, USA | Korean Americans with high chronic HBV infection rates, contributing to liver cancer incidence and mortality. | Involved |
| Masi, 2003, USA | Residents of Chicago's Austin community facing high unemployment, poverty, and low birth weight rates, indicating significant health disparities. | Shared Leadership |
| Mayer, 2019, USA | Low-income residents of East Harlem with pre-diabetes and obesity, focusing on diabetes prevention. | Collaborate |
| Mehta, 2017, USA | People in underserved communities at high risk of or diagnosed with depression, addressing mental health inequities. | Shared Leadership |

| **Author, Year** | **Brief Description** | **Levels of Engagement** |
| --- | --- | --- |
| Andrews, 2016, USA | African American women living in subsidized government housing with high smoking prevalence. | Collaborate |
| Angell, 2003, USA | Women with primary breast cancer living in rural and isolated communities in California, addressing breast cancer survivorship. | Collaborate |
| Celano, 2012, USA | Low-income African American children and their families facing high asthma morbidity and mortality rates. | Outreach |
| Choi, 2016, USA | American Indians/Alaska Natives living in rural or reservation-based areas with the highest smoking prevalence among any racial/ethnic group in the U.S. | Shared Leadership |
| DeHaven, 2011, USA | African Americans living in areas with high poverty, female-headed households, and unemployed males, experiencing higher morbidity and mortality rates. | Shared Leadership |
| Derose, 2014, USA | Residents of Los Angeles evaluated for park-to-citizen ratio and its impact on health outcomes. | Shared Leadership |
| Froelicher, 2011, USA | Low-income African American smokers facing higher age-adjusted mortality rates from tobacco-related cancers and cardiovascular disease. | Consult |
| Goldfinger, 2012, USA | Black and Latino populations impacted by stroke with suboptimal stroke recurrence rates and prevention strategies. | Shared Leadership |
| Ingraham, 2017, USA | Overweight or obese lesbian and bisexual women aged 40 and older with higher body weight than heterosexual women, addressing obesity and mindfulness. | Shared Leadership |
| Islam, 2013, USA | NYC Korean population with a higher risk of diabetes compared to the general population, focusing on diabetes prevention. | Shared Leadership |
| Jernigan, 2018, USA | Native American communities from Chickasaw and Choctaw Nations with obesity, diabetes, and hypertension exceeding national averages. | Consult |
| Kaholokula, 2012, USA | Pacific Islanders with high obesity prevalence and associated health risks. | Shared Leadership |
| Kneipp, 2012, USA | Women in the Temporary Assistance for Needy Families (TANF) program experiencing higher rates of depressive disorders, PTSD, and poor general health. | Collaborate |
| Larkey, 2012, USA | Multicultural and underinsured individuals with low participation in cancer prevention research programs. | Collaborate |
| Lee, 2014, USA | Vietnamese Americans facing high incidence of colorectal cancer and low screening rates, addressing cancer prevention. | Shared Leadership |
| Ma, 2018, USA | Korean Americans with high chronic HBV infection rates, contributing to liver cancer incidence and mortality. | Involved |
| Masi, 2003, USA | Residents of Chicago's Austin community facing high unemployment, poverty, and low birth weight rates, indicating significant health disparities. | Shared Leadership |
| Mayer, 2019, USA | Low-income residents of East Harlem with pre-diabetes and obesity, focusing on diabetes prevention. | Collaborate |
| Mehta, 2017, USA | People in underserved communities at high risk of or diagnosed with depression, addressing mental health inequities. | Shared Leadership |
| Mullany, 2012, USA | American Indian teen mothers facing health inequities including drug abuse, obesity, and intentional and unintentional injuries. | Shared Leadership |
| Nickell, 2019, USA | Breast cancer patients in the San Francisco Bay Area from low socioeconomic backgrounds with limited access to health research participation. | Outreach |
| Owais, 2011, USA | Mother-infant pairs in Karachi, Pakistan, with low childhood immunization rates and related health outcomes. | Outreach |
| Paskett, 2018, USA | Appalachian communities facing high rates of cancer and obesity, focusing on health education and prevention. | Collaborate |
| Patel, 2019, USA | Racially and ethnically diverse adults in underserved Detroit areas with low insurance rates, addressing healthcare access. | Collaborate |
| Pazoki, 2007, Iran | Women aged 25–64 in Iran addressing cardiovascular disease, a leading cause of death among women. | Involved |
| Rhodes, 2017, USA | Hispanic/Latino gay and bisexual men severely affected by HIV, accounting for two-thirds of new infections in the U.S. | Shared Leadership |
| Schoenberg, 2018, USA | Appalachian residents with low socioeconomic status and poor health indicators needing interventions for healthier behaviors. | Consult |
| Schulz, 2015, USA | Non-Hispanic Black and Hispanic residents with high age-adjusted mortality due to heart disease, focusing on cardiovascular health. | Involved |
| Spencer, 2011, USA | African American and Latino adults with higher illness and mortality burdens from Type 2 diabetes. | Consult |
| Tanjasiri, 2015, USA | Pacific Islander women and their partners with high cervical cancer rates, focusing on prevention and screening. | Shared Leadership |
| Wilcox, 2013, USA | African Americans with high morbidity and mortality rates and risk factors for cardiovascular diseases. | Involved |
| Wilson, 2019, USA | Black heterosexual men in Brooklyn, living in socioeconomically disadvantaged areas with high HIV risk. | Collaborate |
| Woods, 2013, USA | African Americans addressing obesity and associated chronic disease risk factors through wellness programs. | Consult |
| Wright, 2014, USA | Inner-city Hispanic/Latino elementary children at high risk due to lack of physical activity, focusing on fitness interventions. | Shared Leadership |
| Zoellner, 2014, USA | African American residents in Hattiesburg addressing high cardiovascular disease prevalence and physical inactivity. | Shared Leadership |
