# Supplementary material for: Evaluation of a large language model (ChatGPT) versus human researchers in assessing risk-of-bias and community engagement levels: a systematic review use-case analysis
Source: Eur J Public Health. 2025 Jun 10;35(6):1082–6. doi: 10.1093/eurpub/ckaf072 (PMC12707484; doi:10.1093/eurpub/ckaf072)
Supplement: ckaf072_Supplementary_Data [file ckaf072_supplementary_data.zip › ejph-2024-09-om-0640-File005.docx]

**Supplementary file 3. Overall and single-domain (from domain 1 to 5) ROB GPT’s and LOER GPT’s judgments and the research team’s (RES).** GPT1 to GPT5 refer to ROB GPT’s judgments for domain 1 to 5, whereas RES1 to RES5 refer to the research team’s risk-of-bias judgments for domain 1 to 5. GPT overall and RES overall refer to the final overall risk-of-bias study judgment. GPT LOER and RES LOER refer to LOER study judgment by LOER GPT and the research team respectively. Regarding risk classification meaning, LR: Low risk, SC: Some concern; HR: High risk; Regarding levels of engagement meaning, OUT: Outreach; CON: Consult; INV: Involve; COL: Collaborate; SHL: Shared leadership.

| **Study** | **GPT1** | **GPT2** | **GPT3** | **GPT4** | **GPT5** | **GPT overall** | **GPT LOER** | **RES1** | **RES2** | **RES3** | **RES4** | **RES5** | **RES overall** | **RES LOER** |
| --- | --- | --- | --- | --- | --- | --- | --- | --- | --- | --- | --- | --- | --- | --- |
| Andreae 2012 | LR | LR | LR | LR | LR | LR | COL | LR | LR | LR | LR | LR | LR | COL |
| Andrews 2016 | LR | LR | LR | LR | LR | LR | COL | LR | LR | LR | LR | SC | SC | COL |
| Angell 2003 | LR | LR | LR | LR | LR | LR | COL | LR | LR | SC | LR | LR | SC | COL |
| Celano 2012 | LR | LR | LR | LR | LR | LR | COL | LR | SC | LR | SC | LR | SC | OUT |
| Choi 2016 | SC | SC | HR | SC | SC | HR | COL | SC | SC | SC | SC | SC | HR | SHL |
| DeHaven 2011 | SC | LR | SC | LR | SC | SC | SHL | SC | LR | LR | HR | LR | HR | SHL |
| De Rose 2014 | LR | LR | LR | LR | LR | LR | COL | LR | LR | LR | LR | LR | LR | SHL |
| Froelicher 2011 | SC | LR | SC | LR | LR | SC | COL | LR | HR | LR | HR | LR | HR | CON |
| Goldfinger 2012 | LR | LR | SC | LR | LR | SC | COL | LR | LR | LR | LR | LR | LR | SHL |
| Ingraham 2017 | LR | SC | LR | LR | LR | SC | COL | LR | SC | LR | SC | LR | SC | SHL |
| Islam 2013 | LR | LR | SC | LR | LR | SC | COL | LR | LR | SC | LR | LR | SC | SHL |
| Jernigan 2018 | LR | SC | LR | LR | LR | SC | COL | LR | SC | SC | LR | LR | SC | CON |
| Kaholokula 2012 | LR | SC | SC | LR | LR | SC | COL | LR | LR | HR | LR | LR | HR | SHL |
| Kneipp 2011 | LR | LR | SC | LR | LR | SC | COL | LR | LR | SC | SC | LR | SC | COL |
| Larkey 2012 | SC | HR | HR | HR | SC | HR | COL | SC | HR | HR | SC | LR | HR | COL |
| Lee 2014 | SC | SC | LR | SC | SC | SC | COL | SC | LR | LR | SC | LR | SC | SHL |
| Ma 2018 | LR | LR | LR | LR | LR | LR | COL | SC | LR | LR | LR | LR | SC | INV |
| Masi 2003 | LR | LR | SC | LR | LR | SC | COL | SC | HR | HR | LR | LR | HR | SHL |
| Mayer 2019 | LR | LR | SC | LR | LR | SC | COL | SC | LR | HR | SC | LR | SC | COL |
| Mehta 2017 | SC | LR | LR | SC | SC | SC | COL | SC | LR | LR | SC | LR | SC | SHL |
| Mullany 2012 | LR | SC | LR | LR | LR | LR | COL | LR | LR | LR | LR | LR | LR | SHL |
| Nickel 2019 | LR | LR | LR | LR | LR | LR | COL | LR | LR | LR | LR | LR | LR | OUT |
| Owais 2011 | LR | LR | LR | LR | LR | LR | INV | SC | LR | LR | LR | SC | SC | OUT |
| Paskett 2018 | LR | SC | SC | LR | LR | SC | COL | LR | HR | SC | SC | LR | HR | COL |
| Patel 2019 | SC | LR | LR | SC | SC | SC | COL | LR | LR | LR | SC | SC | SC | COL |
| Pazoki 2007 | SC | LR | SC | LR | LR | SC | COL | LR | LR | LR | SC | SC | SC | INV |
| Rhodes 2017 | SC | SC | LR | SC | SC | SC | COL | LR | LR | LR | LR | LR | LR | SHL |
| Schoenberg 2017 | SC | SC | LR | SC | SC | SC | SHL | LR | HR | HR | HR | SC | HR | CON |
| Schulz 2019 | LR | LR | SC | LR | LR | LR | COL | LR | LR | SC | LR | LR | SC | INV |
| Spencer 2011 | LR | SC | LR | SC | LR | SC | COL | LR | SC | LR | SC | LR | SC | CON |
| Tanjasiri 2015 | SC | SC | HR | SC | SC | HR | COL | LR | LR | LR | SC | LR | SC | SHL |
| Wilcox 2013 | SC | SC | HR | SC | LR | HR | COL | LR | SC | SC | LR | LR | SC | INV |
| Wilson 2019 | SC | LR | SC | LR | LR | SC | COL | HR | LR | HR | SC | LR | HR | COL |
| Woods 2013 | SC | SC | SC | SC | LR | SC | COL | LR | LR | LR | LR | LR | LR | CON |
| Wright 2014 | LR | SC | LR | LR | LR | SC | COL | LR | LR | SC | LR | LR | SC | INV |
| Zoellner 2011 | HR | SC | SC | SC | SC | HR | COL | LR | LR | LR | LR | LR | LR | SHL |
